# Digital Solutions to Alleviate the Burden on Health Systems During a Public Health Care Crisis: COVID-19 as an Opportunity

**DOI:** 10.2196/25021

**Published:** 2021-06-11

**Authors:** Sofie H Willems, Jyotsna Rao, Sailee Bhambere, Dipu Patel, Yvonne Biggins, Jessica W Guite

**Affiliations:** 1 DayToDay Health Health Innovators Inc Boston, MA United States; 2 Center for Advancement in Managing Pain School of Nursing The University of Connecticut Storrs, CT United States

**Keywords:** coronavirus, digital health, multiplatform, chat, symptom tracking, well-being, COVID-19, online platform, symptom, monitoring, follow-up

## Abstract

The COVID-19 pandemic has generated unprecedented and sustained health management challenges worldwide. Health care systems continue to struggle to support the needs of the majority of infected individuals that are either asymptomatic or have mild symptoms. In addition, long-term effects in the form of long-lasting COVID-19 symptoms or widespread mental health issues aggravated by the pandemic pose a burden on health care systems worldwide. This viewpoint article considers aspects of digital health care solutions and how they can play an ongoing role in safely addressing gaps in the health care support available from initially and repeatedly overwhelmed providers and systems. Digital solutions can be readily designed to address this need and can be flexible enough to adapt to the evolving management requirements of various stakeholders to reduce COVID-19 infection rates, acute hospitalizations, and mortality. Multiplatform solutions provide a hybrid model of care, which can include mobile and online platforms accompanied by direct clinician input and feedback. Desirable components to be included are discussed, including symptom tracking, patient education, well-being support, and bidirectional communication between patients and clinicians. Customizable and scalable digital health platforms not only can be readily adapted to further meet the needs of employers and public health stakeholders during the ongoing pandemic, but also hold relevance for flexibly meeting broader care management needs into the future.

## Introduction

The COVID-19 pandemic created immediate and long-term challenges for health care systems worldwide. This viewpoint examines the role that digital health care solutions play during the pandemic in safely addressing gaps in the health care support available from initially and repeatedly overwhelmed providers and systems, as well as opportunities to alleviate this burden into the future. Approximately one year into the pandemic, health care providers and systems continue to struggle with not only the care management needs of patients diagnosed with COVID-19, but also the added burden of treatment for long-lasting COVID-19 symptoms (ie, “long COVID”) [1-3] and elevated rates of mental health problems associated with the events of the past year [4,5]. Digital solutions can be readily designed to address this need and can be flexible enough to adapt to the evolving management requirements of various stakeholders. This viewpoint offers a recent historical context and perspective on how digital health technology addresses challenges presented by the COVID-19 pandemic and highlights care management opportunities for digital health solutions beyond this public health crisis.

Although the numbers are continuously changing and vary somewhat per country, initial COVID-19 research from different countries indicated that around 18%-20% of individuals who are infected have moderate to severe symptoms and require medical management [6-9]. The remaining 80% of those infected are either asymptomatic or have mild symptoms. The management of this majority subgroup—the 80% of individuals seeking medical care who had suspected or diagnosed COVID-19 with no or mild symptoms related to the disease—posed a challenge to public health management [10-13] and to stressed health care providers and systems [14-17]. Many symptomatic individuals impacted directly by COVID-19 were not hospitalized, in part due to the allocation of limited hospital resources only to those with the most severe symptoms requiring hospitalization [6,14,18]. Health care resource allocation across acute and outpatient clinical care contexts clearly presented a significant challenge in the early phase of the pandemic, which continues to strain systems of care as infection rates fluctuate.

**Figure 1:**
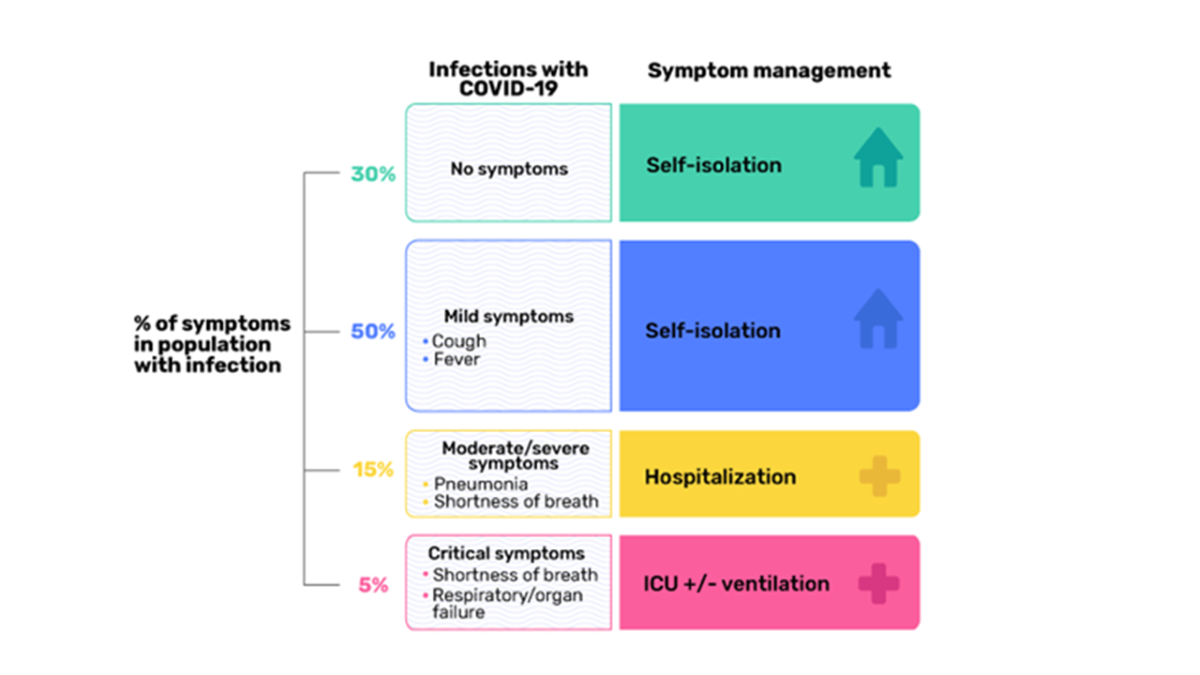
The burden of COVID-19 morbidity and mortality.

An effective public health response to limit the spread of the virus requires those who are suspected of having COVID-19 to be tested to confirm the diagnosis and to take appropriate next steps (Figure 1). The requirement for diagnostic testing in the context of inconsistent availability of testing resources presents another layer of challenge, especially in resource-poor areas or in places where there are few diagnostic tests available. This initially resulted in large numbers of individuals who were suspected of exposure yet unable to receive timely testing, necessitating self-isolation. In addition, individuals who were positively diagnosed, but were either asymptomatic or experienced only mild to moderate symptoms, were advised to self-isolate at home with close and regular follow-up to monitor for any changes in their symptoms. 

The infection rates among health care professionals rose in a magnitude similar to or greater than that observed in the general public [14]. Important information characterizing morbidity among essential workers at risk for COVID-19, including those health care personnel most at risk for mortality [19], emerged to support the need for continued surveillance and the development of strategies to protect essential workers and those they serve. Further underscoring this need was the imbalance between supply and demand for medical resources in many countries, which presented global questions about fair allocation of medical resources and personnel during the pandemic [6]. Factors serving to limit the pool of available health care workers included adherence to public health standards that ideally require an exposed health care worker to quarantine for 14 days. Emerging information about the risk of transmission from presymptomatic cases underscores a sustained need for vigilance, as presymptomatic individuals have an incubation period of 2-14 days, with an average of 4-5 days before COVID-19 symptoms appear and can reportedly infect 1.4-6.5 individuals during that time [20-23]. Potential provider shortages continue to be of concern during periods of high infection levels and corresponding spikes in the number of hospitalizations due to positive COVID-19 diagnoses and related diseases [16].

Such provider shortages initially necessitated reactivation of retired health care workers and onboarding of new medical personnel. For example, many US states loosened their licensing rules to give those with clinical skills the ability to participate, such as allowing out-of-state physicians to practice and requesting retired physicians to volunteer [16]. The governor of New York activated retired and student health care professionals from >52,000 volunteer health professionals, which included 2400 nurse practitioners and 2265 physicians [17]. The US Department of Veteran Affairs recruited retired federal health care workers through social media. Internationally, the Taipei Centers for Disease Control and Prevention responded to the shortage by implementing an ongoing program to recruit and train general practitioners, retired medical professionals, and school nurses [15].

Rising rates of the disease and corresponding increases in the workload of health care personnel resulted in widespread burnout related to the escalating number of hours physicians were working without breaks [24]. In addition to treating patients in the hospital, clinicians were required to follow up on patients who were at home [12]. During this time, the mental health of health care workers was of major concern due to the effects of acute and chronic stress [19,25]. In the absence of a well-organized strategy to sustain the health care work force, scientists and clinicians called for a shift to a longer-term, more sustainable solution [26].

The uncertainty of the COVID-19 disease progression rapidly changed the landscape of the pandemic in several countries. For example, in Italy, where the burden of mortality was for a period of time relatively greater than in other countries, health care professionals perceived the response to COVID-19 as a continuously evolving process with no visible end point. In many medical settings, adapting to changes was required by the hour [27]. As the pandemic continues, new challenges have emerged, such as the management of patients experiencing “long COVID,” with symptoms lasting longer than the acute infection [1,2,28]. The incidence, prevalence, and precise nature of long COVID is not entirely clear as yet [3], nor is the long-term psychological burden of COVID-19 on society as a whole. This is especially the case for patients that spent time in the intensive care unit (ICU). It is thought that more than half of ICU survivors suffer from post–intensive care syndrome (PICS), which is characterized by various symptoms including physical strength deficits, cognitive decline, and mental health disturbances observed after discharge from critical care that persist for a protracted amount of time [29,30].

According to the American Psychological Association (APA) Stress in America 2020 report and APA one-year pandemic update [4,5], there is a steadily escalating national mental and behavioral health crisis due to COVID-19. This includes negative behavioral health outcomes including unhealthy weight gain and increased drinking [4]. Nearly 1 in 5 adults (19%) say their mental health is worse than it was at this time last year and this is particularly true for the youngest generation of adults [5]. By generation, 34% of Gen Z adults report worse mental health, followed by Gen X (21%), millennials (19%), baby boomers (12%), and older adults (8%).There is a similar trend in other countries, along with notable disruptions in mental health services in most countries [31]. Overall, the psychological toll of chronic uncertainty [19] and countless other factors have created a demand for flexible solutions for disease surveillance and follow-up that could be used by workplaces and health care professionals, as well as the general public.

## The Opportunity to Leverage Digital Health Solutions

The novel health care challenges emerging from the COVID-19 pandemic demanded new health care models and patient care management modalities, with digital health strategies holding great potential for delivering solutions [32]. Recent research supports how digital approaches in general can be effectively used to optimize patient care management for individuals across the life span, with attention paid to the unique developmental and psychosocial needs of adult, child, adolescent, and parent/caregiver populations, as well as those living in multigenerational family contexts [33-35]. There is emerging evidence that mobile phone apps are a feasible and acceptable way of administering health interventions for a range of chronic and acute conditions, and can possibly lead to increased patient self-management, health-related behavior change, a reduction in the use of health care resources [36,37], and improved physical and mental health outcomes [38-40]. However, most studies only showed a modest effect [38,40-43], with more and larger studies being required to determine efficacy and establish evidence for best practices. Finally, there is a small but growing economic evidence base focusing on the value of mobile health (mHealth) interventions, suggesting that they can be cost-effective, economically beneficial, and/or cost saving [44,45].

At the onset of the pandemic, there were many pre-existing popular digital apps available that could alleviate the mental health impact of the pandemic for the general public. These apps could conceivably support resilience through meditation or counseling, as well as support socially distanced care access from a health care provider through telebehavioral health. As literature with a focus on COVID-19–specific digital health solutions began to emerge, it was clear that most initial digital health solutions were oriented to surveillance and symptom tracking [46]. However, at the outset of the pandemic, there were no comprehensive digital health care pandemic control strategies identified that could address the needs of the overwhelming majority of individuals impacted by COVID-19. Solutions that could effectively support and follow up on individuals who required self-isolation due to suspected or diagnosed COVID-19 with no, mild, or moderate symptoms was identified as an important unmet need at the outset of the pandemic. As the pandemic continues into its second year, the need to support this large population of individuals who require self-isolation persists in parallel with new public health management phases that are unfolding, focusing on vaccine administration. Thus, digital health solutions continue to serve an important role in meeting the continued management needs of earlier phases of the pandemic while also simultaneously adapting to meet newly unfolding pandemic needs into the foreseeable future**.**

## Digital Health Adaptation to Evolving Pandemic Needs

As pandemic management strategies begin to accommodate the availability of promising vaccines [47-51], the ongoing management of individuals not yet vaccinated and those who remain at risk for acquiring and transmitting SARS-CoV-2 and its new variant strains persists. This new phase of pandemic management faces many challenges, including limited and inconsistent vaccine availability as well as health inequity factors [52] that present further barriers to achieving local, national, and global vaccination goals [49,51,53]. At the individual level, behavioral factors are critical to understanding an individual’s choice to receive the vaccine or not and encouraging desired behavioral follow-through [54]. There is much to learn from behavioral scientists about critical elements to facilitate successful widespread trust and acceptance of the vaccine and the necessary follow-through for individuals to receive required vaccine doses [53,55]. These are just a few of the barriers to overcome to successfully relax COVID-19 precautions and begin to safely resume valued aspects of everyday life that were put on hold due to COVID-19.

A recently published viewpoint article by Laur and colleagues [56] provides an excellent case study of the challenges of building health services in the context of the rapidly changing COVID-19 landscape. The authors offer perspective through lessons learned in adaptive leadership, drawing upon their experience of developing a COVID-19 remote monitoring program, from a hospital-based health system perspective. They draw upon the process of making “pivots” during development, a process that is commonly used by digital health startups to manage uncertainty, and explain how these strategies hold relevance for health care leaders at any time. The viewpoint by Laur et al [56] provides an excellent consideration of the decisions made by hospital-based health service providers developing a COVID-19 remote monitoring plan and this viewpoint provides readers with additional considerations in the digital health development process.

## Development of a Digital Health Solution

The key to a successful strategy for pandemic preparedness and response management is a well-planned, effectively communicated and coordinated emergency response that draws on medical mobilization. Digital health solutions are in many ways an ideal answer to meeting this need. However, they require time and resources to develop, implement, and evaluate fully. The ongoing management of COVID-19–related patient needs continues and a focus on designing a multiplatform digital health care app for health care providers to effectively and safely monitor individuals outside of the hospital setting continues to be important. Central to the process of developing a digital health solution is to ground and inform further decision making in user experience and evidence-based clinical research that speak to the importance and value of including direct input from patients and other key stakeholders to better understand their wants and needs for the digital solution. Both engagement with and adherence to use of a digital health app are critical factors to consider, particularly when there is a goal to demonstrate direct and/or indirect relationships between a patient’s engagement and adherence and a desired outcome (ie, intervention effectiveness). Emerging digital health research provided insights into patient engagement [57,58] and technology-based features and strategies for promoting adherence [59,60].

Additional clinical expert input on design and development should be elicited and include the perspectives of physicians, physician assistants, and nurses. The development process also should elicit patient and caregiver input, with this feedback incorporated at various stages of development. This user experience research should be used early in the development process and include contextual, qualitative research methods to understand patients’ perspective and concerns, as well as clinical, functional, and emotional needs during each phase of the patient journey. This information helps to inform and improve the patient-as-a-customer digital experience and provides a richer understanding of how patients both experience and engage with the developing app. Qualitative interviews with app users can also provide additional details about a patient’s understanding of app content, its ease of use, and his/her emotional responses to the product. This information, in turn, can be used to iteratively adapt and modify the product to improve these features and to ensure both short- and longer-term engagement with the app occurs. In addition, the documentation of patient engagement and adherence information provides an important foundation for future, larger scale clinical research projects that can prospectively test the effectiveness of a digital health intervention and related research hypotheses of interest.

Clinical objectives for the design of a solution that could flexibly support social distancing guidelines intended to reduce COVID-19 infection rates, acute hospitalizations, and mortality require many additional considerations. For example, additional design considerations may include the need for it to be used flexibly by actively practicing, retired, or new medical professionals. The use of a remote, digital interface presents minimal risk of infection for health care providers, which could in turn alleviate stress and prevent further burnout. Additionally, flexibility of a digital platform to allow for changes to be readily incorporated to adapt to evolving pandemic care management needs is of great value. A design that can provide a comprehensive digital health management system that includes close safety monitoring and can deploy an escalation protocol if a patient’s symptoms worsen and the patient requires acute management in a hospital setting is important. A digital platform that can be further customized to allow for maximal flexibility to adapt to the specific needs of a particular target population or treatment context is also desirable.

## Consideration of Solution Components and Functionality

### Platform and Staffing Considerations

The capacity to connect an individual (referred to as a patient), who is either asymptomatic with a positive diagnosis or has tested positive with mild-to-moderate symptoms, and their treating clinician is a desirable feature, particularly if symptoms escalate. To accomplish this, a design that includes both a web-based interface and an app for mobile platforms (ie, iOS and Android) is appealing. This would allow a patient to interact directly with the mobile app components on his/her smartphone and also allow for communication with a clinician. To make the monitoring process more efficient for clinicians, a combination of remote patient self-monitoring with planning for stepped levels of clinician supervision can be protocolized. Clinicians can then successfully monitor multiple patients based on patient risk factors and symptom severity, and acuity-based staffing protocols can be flexibly designed to help to ensure that available support resources are appropriately assigned.

### The Mobile App

To best serve the patient and clinician, the mobile app platform available to the patient can contain functionality for symptom management, patient education, well-being support, and communication with their health care provider, among other options. Patients can engage with educational content and well-being support provided in the mobile app, while clinicians are able to stay informed of the patient’s progress through the patient’s entry of vital signs and symptom tracking. This real-time data sharing would allow for timely intervention and management of patient health concerns.

### Symptom Management

Symptom management can include tracking of vital signs including respiratory rate, temperature, heart rate, blood pressure, and oxygen saturation. The recorded values can be tracked over time and be made graphically visible to patients. If a vital sign is out of the appropriate physiological range, feedback is provided to the patient and can also be shared with an assigned clinician, who could then contact the patient by chat or phone. At any time, the patient could also initiate a chat conversation with the assigned clinician. A built-in daily health check can assess COVID-19 symptoms with the aim of determining the severity of the symptoms and the need for emergency medical support. Based on responses, a patient would be recommended to either continue to monitor symptoms, contact their physician, or seek emergency services. The patient could also initiate a chat functionality with an assigned clinician, who would have real-time access to the patient responses. The patients could also be provided with a temporal view of their symptoms, displayed on a symptom tracker graph, to provide perspective on progress over time to facilitate effective self-management.

### Patient Education and Well-being Support

An app can be designed to include a comprehensive library of educational content for patients to access to inform themselves about COVID-19 symptoms, manage symptoms, and to support overall well-being. Content that includes evidence-based stress reduction strategies, such as mindfulness and relaxation exercises that are effective in reducing patient stress levels, can be accessible through the app [61-63]. Relaxation exercises can be presented through text, image, and video formats. These strategies should be easy to access, and are worthwhile to include as their benefits include reducing the amount of adrenaline and cortisol in the body, which are elevated in many COVID-19 patients; there are also longer term benefits when such strategies are used for dealing with stress and anxiety [64]. Even during extreme stress, exercises focused on breathing and relaxation can alleviate negative thoughts, moods, and feelings, and increase rates of recovery [65]. Additional self-management strategies including progressive muscle relation exercises, guided visualization, and a loving kindness–themed meditation can be included to support improved mental well-being [66,67]. Additional mental health screening and targeted psychological support services may also be integrated into the platform. Having this included can facilitate patients’ ability to smoothly and easily engage in psychotherapeutic contact with an appropriately licensed behavioral health clinician (eg, a psychologist) when a higher level of support is indicated.

### Patient-Clinician Communication

Secure, bidirectional chat communication between a patient and clinician can be initiated 24/7 through the app by the patient or through a web-based portal by the clinician. The clinician response team may be designated by a hospital or a single clinician. Once a patient initiates the chat communication functionality, the assigned clinician will receive a message and would be expected to respond within a predetermined time duration. The clinician may assess the patient’s data and all data input from the patient’s app-based interface in order to provide an appropriate response to the patient’s query. Communication features can further support sharing images, documents, and links, as needed, and can include notifications and reminders to further enhance adherence to treatment recommendations.

## Conclusion

This viewpoint provides additional perspective on how digital health solutions can be used to remotely meet the ongoing challenges that the COVID-19 pandemic presents to health care systems. Multiplatform mHealth/eHealth solutions provide an example to ground and further enrich consideration of how to best meet the evolving needs of a large, significant health care management population. Solutions designed to support the needs of both patients and clinicians safely and remotely for continued follow-up of individuals with COVID-19–related symptoms will remain necessary for the foreseeable future. Moreover, these solutions provide opportunities to flexibly support care management needs of patients, providers, and health care systems beyond this public health crisis.

Components to consider leveraging in a multiplatform digital health care solution designed to connect patients to clinicians for continued follow-up needs are discussed and can safely manage and prevent disease progression and mortality. The solution implemented should align with evidence-based recommendations such as the third domain of the Centers for Disease Control and Prevention’s 2017 Pandemic Influenza Plan [13]. Domain 3 of this plan specifies medical countermeasures to increase access and use of critical countermeasures for response activities. Recently emerging literature speaks to how valuable mHealth systems and platforms that can facilitate access to mobile care providers through telehealth will continue to be for situations requiring self-isolation [46]. Moreover, there is broad-reaching potential for the utility of mHealth systems and platforms as effective solutions for future care management needs beyond the pandemic. The continually increasing widespread adoption of mobile phones worldwide presents significant opportunities for health-related apps to impact health behaviors globally, particularly in low- and middle-income countries [68].

Most solutions at the start of the pandemic were mainly intended to support contact tracing and symptom monitoring for self-use by individuals, without any input from clinicians [46], or remote monitoring solutions created by hospitals and health service organizations adapting to rapidly address shifting institutional needs [56]. The involvement of clinicians and close monitoring of symptoms is critical for individuals with asymptomatic, mild, or moderate disease from the standpoint of the potential for the sudden emergence of severe symptoms and unexpected deterioration [69]. COVID-19–associated acute and long-term health management needs, along with continued adherence to recommended social distancing practices more generally, support the lasting value of similar digital solutions into the future.

While additional research is always needed to test the efficacy of a newly developed intervention, the urgency of the pandemic demanded a need to rapidly develop and implement digital solutions. Researchers are highlighting the need for the development of hybrid digital health solutions and models of care that combine mobile and online platforms with direct clinician input, while also addressing the needs of impacted patients across the developmental continuum [33-35,70]. Future research efforts should take steps to evaluate the efficacy of these interventions as well as the economic impact of an mHealth intervention with respect to its cost-effectiveness to add to the limited evidence base that currently exists [44,45].

The adaptability of digital platforms to flexibly accommodate various components and content can not only reduce the significant patient-care burden experienced by health care professionals, but also can be useful in other settings. For example, these digital platforms can be customized to support the needs of an employer’s management of employee symptoms or the public health needs of a government continuing to combat and effectively manage the current and future phases of the COVID-19 pandemic. Ultimately, the rapid adoption of mobile technology globally [68], coupled with the adaptability of digital health platforms and their content, provides valuable opportunities for improved health care throughout the pandemic and beyond.

## Abbreviations

APA: American Psychological Association
ICU: intensive care unit
mHealth: mobile health
PICS: post–intensive care syndrome
